# Older Adults Perceptions of Technology and Barriers to Interacting with Tablet Computers: A Focus Group Study

**DOI:** 10.3389/fpsyg.2017.01687

**Published:** 2017-10-04

**Authors:** Eleftheria Vaportzis, Maria Giatsi Clausen, Alan J. Gow

**Affiliations:** ^1^Department of Psychology, School of Social Sciences, Heriot-Watt University, Edinburgh, United Kingdom; ^2^Division of Occupational Therapy and Arts Therapies, School of Health Sciences, Queen Margaret University, Edinburgh, United Kingdom; ^3^Centre for Cognitive Ageing and Cognitive Epidemiology, University of Edinburgh, Edinburgh, United Kingdom

**Keywords:** aging, older adults, tablet computers, technology, focus groups, qualitative

## Abstract

**Background:** New technologies provide opportunities for the delivery of broad, flexible interventions with older adults. Focus groups were conducted to: (1) understand older adults' familiarity with, and barriers to, interacting with new technologies and tablets; and (2) utilize user-engagement in refining an intervention protocol.

**Methods:** Eighteen older adults (65–76 years old; 83.3% female) who were novice tablet users participated in discussions about their perceptions of and barriers to interacting with tablets. We conducted three separate focus groups and used a generic qualitative design applying thematic analysis to analyse the data. The focus groups explored attitudes toward tablets and technology in general. We also explored the perceived advantages and disadvantages of using tablets, familiarity with, and barriers to interacting with tablets. In two of the focus groups, participants had previous computing experience (e.g., desktop), while in the other, participants had no previous computing experience. None of the participants had any previous experience with tablet computers.

**Results:** The themes that emerged were related to barriers (i.e., lack of instructions and guidance, lack of knowledge and confidence, health-related barriers, cost); disadvantages and concerns (i.e., too much and too complex technology, feelings of inadequacy, and comparison with younger generations, lack of social interaction and communication, negative features of tablets); advantages (i.e., positive features of tablets, accessing information, willingness to adopt technology); and skepticism about using tablets and technology in general. After brief exposure to tablets, participants emphasized the likelihood of using a tablet in the future.

**Conclusions:** Our findings suggest that most of our participants were eager to adopt new technology and willing to learn using a tablet. However, they voiced apprehension about lack of, or lack of clarity in, instructions and support. Understanding older adults' perceptions of technology is important to assist with introducing it to this population and maximize the potential of technology to facilitate independent living.

## Introduction

Technology now supports or streamlines many day-to-day activities. This continued technological development is occurring alongside the aging of global populations, creating opportunities for technology to assist older people in everyday tasks and activities, such as financial planning and connecting with friends and family. New technology also has the potential to provide timely interventions to assist older adults in keeping healthy and independent for longer (Geraedts et al., 2014). Older adults are slower to adopt new technologies than younger adults (Czaja et al., 2006), but will do so if those technologies appear to have value, for example in maintaining their quality of life (Heinz et al., 2013). To make technology more age-friendly, it is important to understand the advantages and disadvantages that older adults perceive in using it. We therefore explored older adults' familiarity with and barriers to using technology.

Mobile technological devices such as tablet computers (commonly referred to as tablets), a type of portable computer that has a touchscreen, are becoming increasingly popular. The number of adults aged 65–74 years using tablets to go online more than trebled in recent years in the UK, going from 5% in 2012 to 17% in 2013. However, this percentage remains low compared with younger age groups (e.g., 37% of adults aged 25–34 years used tablets to go online in the last 3 months) (Ofcom, 2014a). Adoption of technology may improve older adults' quality of life, facilitate independent living for longer (Orpwood et al., 2010), and bridge the technological gap across generations by teaching older people to use technological devices (Bailey and Ngwenyama, 2010). Tablets can offer the same functionality as a normal computer at a smaller, more flexible size and weight. Tablets may also provide a better internet browsing experience compared to mobile phones as they have a larger screen. According to Ofcom (2014b), tablets helped to drive overall internet use in adults over 65 from 33% in 2012 to 42% in 2013. Older adults may prefer tablet technology due to the portability and usability they provide vs. computer technology (e.g., adjustable font or icon size), especially to those who have a wide range of motor and visual abilities (Chan et al., 2016). Understanding the barriers to using technology in general and tablets in particular in older adults can provide insights into appropriate ways of introducing tablet technology to this population. This is important as it appears that tablet technology encourages older adults to access the internet. In turn, this may assist in daily activities and decrease isolation, which is more common in older age (Cornwell and Waite, 2009). The internet may foster links to friends and family and facilitate essential daily activities, such as shopping and banking (Czaja et al., 2006).

Previous studies have explored the perceptions and attitudes of older adults toward new technologies. Heinz et al. (2013) conducted focus groups with 30 older adults in total (mean age 83), focussing on daily needs and challenges, advantages and disadvantages associated with technology usage, how technology could be helpful, and ways to make technology easier to use. Participants were apparently willing to adopt new technologies when their usefulness and usability surpassed feelings of inadequacy, though some concerns remained over society's overreliance on technology, loss of social contact, and complexity of technological devices. Mitzner et al. (2010) conducted 18 focus groups with 113 community-dwelling older adults (mean age 73 years). Participants reported using technology at home, at work and for healthcare. Positive reactions to technology included portability and communication, whereas too many options and unsolicited communication were seen as disadvantageous.

The Center for Research and Education on Aging and Technology Enhancement (CREATE) has also reported on the use of technology among community-dwelling adults. Their findings suggested that older adults (60–91 years) were less likely than younger adults to use technology in general, and specifically computers and the internet. Technology adoption was associated with higher cognitive ability, computer self-efficacy and computer anxiety, whereas higher fluid intelligence and crystallized intelligence predicted the use of technology; higher computer anxiety predicted lower use of technology (Czaja et al., 2006). An earlier study indicated that older people (60–75 years) perceived less comfort, efficacy and control over computers relative to younger participants, however, direct experience with computers resulted in more positive attitudes (Czaja and Sharit, 1998). Alvseike and Brønnick (2012) reported that cognitive deficits and low self-efficacy associated with older age significantly reduced participants' ability to use technology. Generally, the current literature suggests that although older adults are open to using technology there may be age-related (e.g., cognitive decline) as well as technology-related (e.g., interface usability) barriers.

Tablets offer less complexity compared with other operating systems as they comprise a touch-based interface. For example, Umemuro (2004) developed an email terminal with a touchscreen and compared it with the same terminal using a standard keyboard and mouse in two groups of Japanese adults (60–76 years old). Participants were required to read and send messages using their assigned terminals. Results suggested that participants using the touchscreen terminal were less anxious compared with those using the standard keyboard terminal. Schneider et al. (2008) also compared different input devices including a touchscreen with a mouse, or eye-gaze plus keyboard, in sample ranging from 60 to 72. Participants were required to click inside a start stimulus (circle) and then inside a target (square) that appeared on a screen, or to move a stimulus (rectangle) toward a target (another rectangle) on a touchscreen. The authors concluded that the touchscreen input afforded the best performance as reflected by execution time, error rate and subjective evaluation of task difficulty. Interestingly, the older participants (60–72 years) reached a performance level similar to that of younger participants (20–39 years) when using a touchscreen; that is, while they remained slower, this was no longer significantly different. Although the design of applications running on devices is critical, touchscreen interfaces may make it easier for users to complete tasks and contributes to the popularity and success of touchscreen devices (Balagtas-Fernandez et al., 2009).

The overall aim of the current study was to build on previous research by investigating the perceptions of, and barriers to, interacting with tablets in healthy older adults who were novice tablet users. We employed focus groups as this methodology offers an open and exploratory way for qualitative data collection (Krueger, 1998). We wanted to understand: (a) older adults' attitudes toward technology in general, and tablets in particular; (b) the perceived advantages and disadvantages of using tablets, and how they may be helpful; and (c) familiarity with, and barriers to interacting with tablets. These objectives were conceived so that we might harness user-engagement to refine protocols from previous research in which tablet training has been used as a cognitive intervention (Chan et al., 2016), thus directing our future research efforts.

## Methods

### Participants

Potential participants were recruited from the Edinburgh area by contacting clubs for older adults, community centers and email lists using the snowball principle (Goodman, 1961). All potential participants provided demographic information by telephone, including that they were free of neurological and psychiatric conditions, and cognitive and motor impairment. Eleven potential participants were excluded at this stage because they did not meet these criteria, and one further participant declined for personal reasons. In total, 18 healthy, community-dwelling older adults between the ages of 65 and 76 years (*M* = 71.1; SD = 3.7) agreed to participate in the focus groups. Three focus groups were conducted and each included six participants. All participants were tablet novices, but ranged in their experience with other computing technology (i.e., desktop computers). Those with no previous computing experience were included in one focus group; participants in the two remaining groups all had previous computing experience. The same agenda was used for all groups. Demographic information for the focus group participants is presented in Table 1.

**Table 1:** Demographic characteristics of the participants (*N* = 18).

**Variable**	***N* (%)**
**SEX**	
Female	15 (83.3)
Male	3 (16.7)
**ETHNICITY**	
White British	15 (83.3)
White other	2 (11.1)
Did not respond	1 (5.6)
**EDUCATION**	
Some high school	1 (5.6)
High school	4 (22.2)
Some college	6 (33.3)
Graduate	4 (22.2)
Post-graduate	3 (16.7)
**LIVING STATUS**	
Alone	13 (72.2)
Partnered	4 (22.2)
Carer	1 (5.6)

*Percentages may not total 100% due to rounding*.

### Materials and procedure

We developed focus groups materials based on previous research (Venkatesh et al., 2003; Zhou et al., 2014; Chan et al., 2016). A number of different devices were made available to the participants during the second half of the focus groups to gain feedback from older adults on any likely preferences for size, style, etc., to better direct the selection of the tablet device to be used in a later planned intervention study. We chose the following five touchscreen tablets using independent advice and product reviews at www.which.co.uk: Asus TF103CX (10″, Android), Asus Google Nexus 7 (7″, Android), Samsung Galaxy Tab 3 (8″, Android), Samsung Galaxy Tab 3 (10.1″, Android), and Apple iPad Mini (7.9″, iOS). We covered the brand names/logos on all tablets with masking tape. A previous study investigating text entry on tablets and smartphones in older Chinese adults used four different touch screens: Apple iPod Touch, Dell Streak, Samsung Galaxy Tab and Apple iPad (Zhou et al., 2014).

We conducted the focus group sessions between February and March 2015 in a quiet room at Heriot-Watt University, Edinburgh. These focus groups were the first stage of a larger study, “Tablet for Healthy Ageing” (Vaportzis et al., 2017). The focus group stage was designed to utilize user-engagement to explore older adults' perceptions and attitudes toward tablets and technology in general, and also to refine a proposed intervention protocol using technology with older adults for subsequent stages of the “Tablet for Healthy Ageing.” During the focus groups, participants were specifically asked to provide feedback on the proposed intervention protocol, which referred to themes and activities that might appear during a tablet training course. The group discussions lasted ~2 h, and the same moderator, who was one of the authors of this study (E.V.), conducted all focus groups. Participants were seated around a table with the moderator being seated with them at the table.

Before beginning the focus groups, the moderator reminded participants of the objective of the study, and that the discussions would be used to guide the next stage of the research. Participants gave written informed consent and completed a brief demographics questionnaire reported in Table 1. To ensure anonymity, participants' responses could not be linked with participants' identities. Below we present the questions that guided and stimulated the discussion over the first hour.

- Think for a moment about your daily life. What are some of the greatest needs and challenges you have?
- What is technology for you? What does the word “technology” bring to mind?
- Which technologies do you use?
- Given some of the issues that people your age face that we just discussed earlier (such as [examples from earlier conversation]), which technologies do you know about that might be helpful in addressing these problems?

The moderator then pointed toward a selection of tablets, which were arranged on an adjacent table though not switched on, and asked the participants:

- Have you seen a tablet before?
- What are some of the reasons for which you have not used a tablet to date?
- What do you think are the advantages using tablets?
- What do you think are the disadvantages using tablets?

The moderator handed out the tablets. The tablets remained turned off as the main interest at this point was participants' first impressions on the physical aspects of the tablets such as weight and size. Participants took turns to get a feel for all different models. As there were five tablets but six participants, in any given activity two participants shared a device. Different participants paired up for the various tasks to ensure that each participant had the opportunity to complete some of the tasks on their own.

The moderator asked then the following questions:

- What are your initial impressions of the tablets? What is the first thing that comes to your mind?
- How can a tablet be helpful in:
  - Assisting with everyday living
  - Improving mental abilities
  - Improving general health and wellbeing

After a break, the second hour comprised an interactive session. The moderator gave instructions on how to turn the tablets on, and participants used three applications (apps) in the following order: Google Maps, BBC News, and Chrome browser. We used a brief scenario for each application, and the scenarios were linked to give participants a realistic sense of how people use tablets in their everyday lives. The scenario involved meeting with a friend at the Scottish National Gallery after the focus group. Participants used the Google Maps app to choose their preferred way to get there from Heriot-Watt University. On arriving at the gallery early, the scenario suggested they accessed the BBC News app. They read the news and watched live TV streams. Finally, once their friend arrived, they used the Chrome browser to find out what was on at their preferred cinema.

Then, the moderator asked:

- What are your impressions of the tablet applications that you used?
- What might make it easier to use a tablet?

The moderator handed out printed copies of a tentative intervention programme that would be used in the following stage of the study (Vaportzis et al., 2017). The programme included topics that would be covered during the 10-week intervention, including social connectivity and traveling and was based on a previous study (Chan et al., 2016). Once participants had enough time to look at the programme, the moderator asked:

- What are your thoughts? What do you think that may or may not work with this programme?

Finally, participants completed a Tablet Experience Questionnaire to rate their experience with the tablets, and give their opinion about the tablets and applications. All focus group sessions were video-recorded and later transcribed verbatim. The moderator did not take notes during the sessions; rather these were transcribed verbatim from the recordings. The full transcripts were then analyzed as detailed below.

This study was approved by the Heriot-Watt University School of Life Sciences Ethics Committee. All subjects gave written informed consent in accordance with the Declaration of Helsinki.

### Data analysis

Data analysis was first conducted by one of the researchers (E.V.) and subsequently by an independent researcher with experience in qualitative data analysis to increase confirmability (M.G.C.). We carried out inductive thematic analysis as described by Boyatzis (1998) using NVivo10 software (NVivo, 2012). The focus groups transcripts were initially read numerous times. This process of immersion with data is thought to serve as a “preparation” stage before the actual analysis as it allows familiarization with the language and wording used by the participants. Initially, first-order themes were identified within each response of each participant to the questions posed in the focus groups. These themes were either directly related to the study's research questions, or were entirely new topics that emerged from the participant's comments.

In the next stage, these first-order themes were fused or clustered to a second-order series of themes (”higher order” themes or codes) based on the commonality of their meaning. At this stage, themes from the previous stage either expanded to encompass others or had “shrunk” to become more specific. These final themes were more abstract in their meaning than the previous ones and, were the themes to be finally interwoven with the existing literature (e.g., “cost” and “lack of instructions and guidance” were fused under “barriers to using technologies and tablets”). The process described above was iterative as themes evolved and the data better understood. Further reading led to the identification of additional themes, initially not detected.

Each of the researchers individually coded and categorized data from the same focus group to allow triangulation of findings. Data from the other two focus groups were then coded by one of the researchers (E.V.), and were reviewed repeatedly with particular attention to refining the codes by both researchers. Through comparison, the two researchers discussed and agreed on discrete themes. We refined and finalized the codes, resulting in a list of agreed themes.

## Results

The analysis of the focus group transcripts revealed an emphasis on the advantages and disadvantages of using technologies in general with a focus on tablet use. The final four themes were: (a) Barriers to using technologies and tablets; (b) disadvantages and concerns about using technologies and tablets; (c) advantages and potential of technologies and tablets; and (d) skepticism and mixed feelings about technology and tablets. The four themes were common to participants that had previous computer experience and participants that had no previous computer experience, though there were some small differences in the subthemes. For example, lack of instructions and guidance was a subtheme that emerged only in the group that had computer experience. The themes are presented in order of their importance determined by frequency and uniqueness. Participants' quotes are presented to illustrate each theme. Group is indicated by a G and participant by a P next to each quote followed by the appropriate number (e.g., G1, P1). To differentiate the quotes provided by gender, those from male participants are denoted with an M. We present first quotes from participants with previous computer experience (G1 and G2), followed by quotes from participants that reported no/minimal computer experience (G3). A summary of the themes and subthemes is presented on Table 2.

**Table 2:** Focus group themes and subthemes.

**Theme**	**Subthemes**
Barriers to using technologies and tablets	Lack of instructions and guidance
	Lack of knowledge and confidence
	Health-related barriers
	Cost
Disadvantages and concerns about using technologies and tablets	Too much and too complex technology
	Feelings of inadequacy and comparison with younger generations
	Lack of social interaction and communication
	Negative features of tablets
Advantages and potential of technologies and tablets	Positive features of tablets
	Accessing information
	Willingness to adopt technology
Skepticism and mixed feelings about technology and tablets	

### Barriers to using technologies and tablets

Participants mentioned a number of perceived and actual barriers to using tablets and technology in general. Four subthemes emerged under this theme: lack of instructions and guidance, lack of knowledge and confidence, health-related barriers, and cost.

#### Lack of instructions and guidance

Participants noted that if there are any instructions, they are too technical.

G2, P1(M): “The manual is written by the techies. It's not written by [users] and that's probably a big message to send to the manufacturer.”
G2, P2: “There might be features [on the tablets] there which might help you from a medical point of view if you knew about them. So you want the manual to be written in the so-called dummy style, so that it's very readable and understandable.”

Participants in another group noted:

G1, P4: “If you're sitting there by yourself trying to read the instructions that would be quite scary.”
G1, P6: “A little handout with each tablet showing what the keys are for.”

Lack of instructions and guidance was a subtheme that did not emerge in the group that had no computer experience.

Several participants mentioned that when they asked for assistance, other people quickly completed the job for them instead of guiding them.

G2, P2: “My daughter comes and helps me, but she does [participant makes quick noise]. There you are mother. And I'm going […] and say, what did your fingers do?”
G2, P3: “I've got a son and I say “How do I do this?”, and he sets it up for me.” A participant with no computer experience also mentioned: “My son is just too fast. He says it's common sense, use your brain, you should know this. They just have no patience […] they expect to tell you once (G3, P3).”

#### Lack of knowledge and confidence

Participants emphasized their concern and fear of using tablets and technology in general due to lack of knowledge or low confidence, as well as the perceived dangers of technological equipment. One participant said: “But does it get our confidence, the fact that we don't know how to do all these fancy things…I feel a bit inadequate sometimes (G2, P4).” Participants' responses also suggested that they were not aware of differences between different types of technology (e.g., tablets vs. computers). “That's why I'm trying to find out what is the difference between A (tablets), B (computers) and C (smartphones), apart from a bit more this and a bit less of that, there doesn't seem to be any difference [G3, P5(M)].” Another participant expressed fear of using technology: “I'm just frightened in case I go in somewhere and then I can't get out. You know how they talk about the Trojan viruses and all that spyware and all the rest of it? That's what I'm frightened of, especially when you don't know (G3, P6).”

#### Health-related barriers

Barriers related to a number of health issues that older people are more likely to have were noted, illustrated by the following quote: “Health is an issue. I mean, I'm quite healthy […] but my knees and arthritis. You can't stop these things and they have an impact on you, how you approach things (G1, P3)”. A participant stated: “Wasn't it a controversy when they produced eBooks that some people found they couldn't read it under certain lighting conditions as well as any problems with eyesight? [G1, P5(M)].”

Participants with no computer experience also mentioned health-related issues.

G3, P1: “I have difficulty reading signs and anything small. So I would automatically go for the biggest tablet.”
G3, P3: “I don't know if I would be able to use this [tablet] for a long length of time; […] with my [fractured] wrist and fingers I don't know.”

#### Cost

The high price of tablets and other technological equipment was one of the barriers that participants mentioned.

G2, P2: “There's also the cost, because you've got software here and you've got software on your main machine and that's always going to be updated every so often […].”
G2, P4: “Cost comes in to it. It's not so bad nowadays, but it came in to it.”
G2, P1(M): “Often with technology, if it's a low price, you've probably got fewer facilities.” Cost was a subtheme that did not emerge in the group that had no computer experience.

### Disadvantages and concerns about using technologies and tablets

Participants noted a number of issues that discouraged them from using tablets and other technology. Four subthemes emerged under this theme: too much and too complex technology, feelings of inadequacy and comparison with younger generations, lack of social interaction and communication, and negative features of tablets.

#### Too much and too complex technology

Participants felt that there are too many pieces of technology: “You can have too much technology; if you've got a phone and a tablet, and a laptop and a computer, you're swimming about in it [G2, P1(M)].” Participants also expressed preferences for simpler forms of technology: “I just want the simplest, no frills, no bells and whistles [piece of equipment] (G1, P1).” A participant with no computer experience also said: “Is there a very simple tablet, where you can just say I only want my tablet to do that, that and that? I don't want a million opportunities flashing up every time I touch something. It's trying to sell me something I don't want. I just want to be able to do ABCD (G3, P4).”

#### Feelings of inadequacy and comparison with younger generations

In several cases, participants compared themselves to younger people who appear to know how to use technology from a very young age.

G1, P1: “My children just look at a piece of equipment and they're off. […] My brain is just not built to deal with half of the technology out there. I wish I was more the other way, I really do, because I am aware of the being left behind I suppose.”
G1, P4: “I think maybe this is the first generation where the younger people have the advantage over the older people, because they grow up with technology at school.”

A participant in another group also agreed: “A lot of people that [young] age, they seem to pick it up intuitively [G2, P5(M)].”

A feeling of inadequacy compared with younger generations was also reflected in the following quote by a participant with no computer experience: “We are now the children in our children's eyes, I think (G3, P6).” Another participant was skeptical about people's overreliance on technology, and whether this is necessary: “I've been on a train […] and everybody you look at is sitting there with a tablet (G3, P3).”

#### Lack of social interaction and communication

Participants expressed concern about the lack of social interaction and social skills of future generations. A participant noted: “I think they're [younger people] missing a lot of human interaction, because they are so focused on the screen and machines (G1, P2).” Similarly, a participant with in another group said: “The oddest for me is looking at neighboring tables in a public place and I think ‘Why are you out with this person? (G3, P1)”’ Another participant said: “Well, I take my grandchildren out, if we go for lunch I take their phones off them, because they were sitting at lunch, and you say look, I come down to see you at Christmas, family lunch, take them out, and they all seem to do this all the time. You know, you've been brought up to sit down and talk with your elders and your betters round this table, your mother and your father are there and I'm here, and I've come to see you, so don't sit and play games [G3, P5(M)].

#### Negative features of tablets

In terms of the perceived disadvantages of using tablets, some participants with computer experience thought that the tablets were quite heavy, for example: “I don't know if I could be bothered carrying that (the tablet) around with me all the time [G2, P5(M)].”

Some found the buttons cumbersome:

G3, P3: “We press one (button) at the bottom here do we? It would help if they just put a little name.”
G3, P5(M): “I think the buttons are far too difficult to handle and they should have a label on them saying what they are.”

### Advantages and potential of technologies and tablets

Overall, participants rated their tablet experience as positive and most stated that they would be likely to use a tablet in the future. Three subthemes emerged under this theme: positive features of tablets, accessing information, and willingness to adopt technology.

#### Positive features of tablets

Participants with computer experience were impressed with the screen clarity of the tablets, as the following comment suggests: “It's very clear, it's a nice clear screen, because I thought maybe being so small I would have difficulty reading it, but this will be fine (G1, P3).” They also stressed how important portability and versatility was.

G2, P2: “One of the advantages […] is how versatile they are. They can play music. […] They take photos […]. They can do anything really, can't they?”

Another participant in the same group said: “My neighbor next door who's in […] the target age group for this project, they've got a PC and they've got this type of device [tablet] in the lounge all the time and is used regularly […] cause I pass the window, I can see them using it, because it's convenient. Don't have to go upstairs to the equipment [G2, P5(M)].”

G2, P3: “I just had a great granddaughter born on Monday and one of the other grandmothers came along with a tablet and she was taking photos with the tablet. It was tremendous and the quality is really good and that's something that, you know, you can't do on the phone, or you certainly can't do on the laptop.”

Positive features of tablets was a subtheme that did not emerge in the group that had no computer experience.

#### Accessing information

Participants with computer experience noted that tablets give easy access to information:

G2, P4: “Information right away. I like that.”
G2, P3: “I'd know whether the bus is gone or it's coming, that's really important.”
G2, P5(M): “I see people going on holiday […] using these things [tablets], cause you pull up maps…”

#### Willingness to adopt technology

Participants expressed interest in learning how to use a tablet as they felt left out.

“I'd really like to be in the modern world and to be able to manage these things and to be able to access more. I just feel very limited in what I'm doing. And I need the courage and to try and trust somebody to give me what I can manage and […] show me how to get in to it (G2, P6).”

Participants in the no computer experience group also stated:

G3, P6: “I think we're missing out on a lot, because all the information is at hand and we don't know how to collect that information. That's what I personally feel and I want to be able to collate it and just basically know what I'm doing.”
G3, P5(M): “I just think we're like a forgotten generation, that's what I feel like. You want to go in and you want to be able to talk with your family and your grandchildren and not look vacant when they say, I'm going to do this.”

### Skepticism and mixed feelings about technology and tablets

Participants were less in agreement regarding the potential of tablets to improve skills and cognitive abilities. Some participants held that learning to use a tablet could improve various skills and abilities.

G2, P5(M): “Yeah, it keeps the brain active in one way or another.”
G2, P4: “For games and that kind of stuff. I'm sure that's why we all do our Sudoku and all these games, things, code words. Yes, because I notice when you stop, you know, on holiday and you haven't access to the paper or whatever, it takes a while to get some of the more complicated words. But, you know, you lose it for a wee while and then you build it up again.”
G2, P1(M): “Learning any new skill, surely is helping the cognitive function.”

A participant in the group with no computer experience also said: “Oh, I think so, definitely, I do [think that a tablet could be used to improve mental abilities] (G3, P6).” However, others were more skeptical about a tablet's ability to improve older people's skills and abilities as the following quotes by participants with computer experience suggest:

G1, P4: “But, do you know what, these kind of technologies actually make it harder to focus.”
G1, P1: “It almost deters you from memory because you've got your calendar on your phone, you don't have to remember any more.” A participant in the group with no computer experience was also skeptical about the ability of a tablet to improve skills and abilities: “What about the reverse of this […] it's stopping you thinking that six sixes are 36 [G2, P1(M)].”

Participants also did not reach consensus about tablet size, as some favored the small tablets due to portability, but others the larger tablets due to the increased screen size.

G2, P4: “I think with me, it would be the portability of it […]. This is probably slightly too big to go in to my handbag, but the smaller one would.”
G2, P6: “Size would matter to me as well, but I would go for the big one. And ease of use.”

Similarly, in the group with no computer experience participants said:

G3, P1: “[I prefer] the smaller one. It's easier to handle. It's lighter”.
G3, P3:“But the thing about the bigger one is […] if you're looking at something like a film you'll get a bigger picture.”

**Figure 1:**
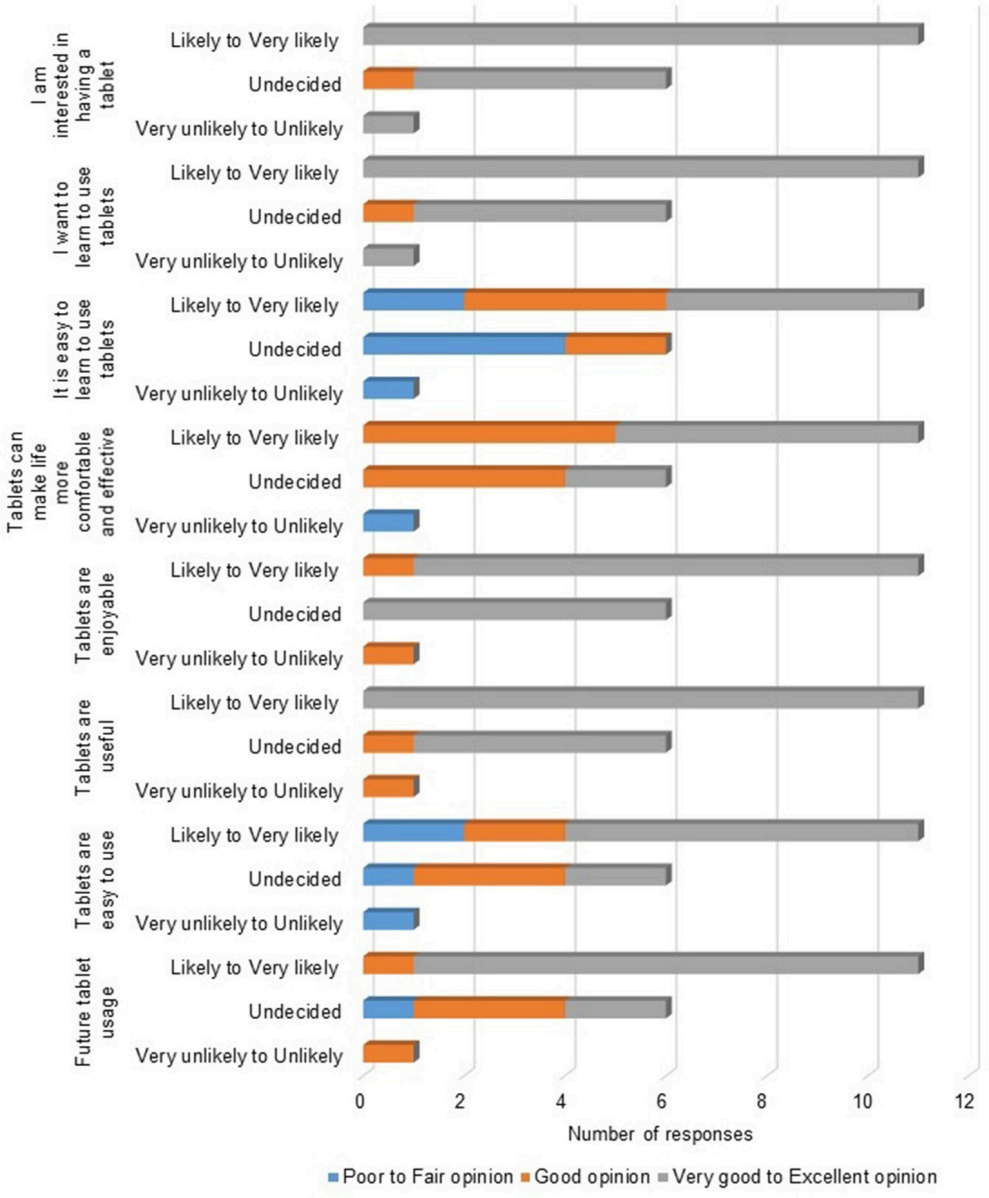
Cross-classification of overall tablet opinion.

After completing the focus groups participants rated their tablet experience on a 5-point Likert scale (1 = Poor to 5 = Excellent). The majority of participants rated their experience Good to Excellent (94.4%) and stated they would be Likely or Very likely to use a tablet in the future (66.6%). Participants reported that they liked the following: access to information (33.3%), tablet size (33.3%), portability (16.7%), screen clarity (11.1%) and versatility (5.6%). They rated as least desirable: not knowing how to use it (27.8%), small buttons and keyboard (27.8%), sensitivity to touch (27.8%) and size (5.6%); 11.1% reported no negative aspects. Interaction among the overall tablet opinions and the various specific variables (e.g., future tablet usage) are presented in a cross-classification figure (Figure 1).

## Discussion

Our qualitative study explored the acceptability and usability of tablets as a potential tool to improve the health and wellbeing of older adults. Our findings supplement previous studies that investigated perceptions and attitudes of older adults toward new technologies (Mitzner et al., 2010). Past research focused on a broad range of technologies, whereas we focused on one specific type of technology (tablets), and therefore incorporated a more hands-on interactive element to the focus groups. Our focus groups considered older adults' views about how they might use tablets as a potential tool to improve their health and wellbeing, in addition to highlighting general attitudes toward technology and tablets, and what might hinder or facilitate using technology and tablets.

The majority of participants enjoyed the tablet experience, and emphasized the likelihood of using a tablet in the future. The positive appraisal of participants' tablet experience was further evidenced by the fact that half of them requested to be included in the following stage of our study. Despite that, the majority of participants lacked confidence in their own abilities to use a tablet. It was also evident from participants' questions that they were unaware of the similarities and differences between different types of technology (e.g., laptop vs. tablet). Overall, participants acknowledged the importance of adopting technology to “move on” and be able to communicate better with younger generations. However, they were concerned about younger people's lack of interaction and communication. Some noted that nowadays people rely too heavily on technology, which is too complicated, and voiced a preference for simpler devices.

Our analyses explored the study's main objectives related to older adults' attitudes toward tablets and technology, the perceived advantages and disadvantages of using tablets, as well as familiarity and barriers to interacting with tablets. In addition to the main objectives of the study, a secondary aim was to refine protocols from previous research in which tablet devices were used as the basis for interventions for cognitive ageing (Chan et al., 2016), to replicate that work in subsequent stages of the “Tablet for Healthy Ageing” research programme. Focus group outcomes confirmed that these protocols used previously with a sample of healthy older adults in the USA were appropriate for a UK sample. Therefore, we did not make any major protocol changes for the planned intervention stages (Vaportzis et al., 2017).

The themes that emerged in the current study were consistent with the literature. For example, disadvantages and concerns about using technologies and tablets emerged both in our study and Heinz et al. (2013), although the latter labeled the theme “Frustrations, Limitations, and Usability Concerns.” In both studies participants noted that tablets and technology in general are often overly complicated and mentioned that simplified technology would be preferable. Concerns about society's overreliance on technology and the perceived growing lack of social interaction and contact were also noted in both studies. Common subthemes with Mitzner et al. (2010) included a fear of using technology, the perception of there being too many options offered by technology, barriers that health issues may impose, as well as the high cost of technological equipment. Interestingly, only participants with previous computer experience brought up the latter in our study. It is possible that cost is not one of the main barriers to using technology for people with less experience; their lack of exposure to technology in general may mean they are less aware of the costs of such devices, or simply that other concerns take priority. For example, Czaja et al. (2006) reported that higher computer anxiety predicted lower use of technology. Although we did not measure anxiety levels, it may be that for some, a lack of confidence rather than cost of equipment is what presents the primary barrier. Another possibility is that the perceived benefits of using technology may outweigh the cost for participants with no computer experience. This finding is consistent with previous studies suggesting the perception of potential benefits was more indicative of technology acceptance than perception of cost (Melenhorst et al., 2001; Mitzner et al., 2010). The theory of diffusion of innovations (Rogers, 2010) also holds that older adults are less likely to adopt new technologies unless they view clear benefits of using them.

Despite the potential barriers and disadvantages of tablets and technology, our findings were also broadly consistent with past research that highlighted their potential advantages. For example, participants in Mitzner et al. (2010) and our study mentioned positive features of tablets and technology, including quick access to information. In addition, in line with Heinz et al. (2013) and Mitzner et al. (2010) our participants indicated that they were eager to adopt technology. Previous studies reported that older adults may be willing to use new technological devices when their usefulness and usability outweigh self-efficacy feelings (Heinz et al., 2013). In addition, it has been suggested that older people with high self-efficacy are less anxious about, and more likely to use, technology in general (Czaja et al., 2006; Mitzner et al., 2010). Our findings appear to be consistent with the suggestion that if older adults were more confident they would be more likely tablet or technology users.

Tablets appear to be user-friendly as they are less complex than other interfaces and do not require wired infrastructure. However, they require support to be introduced to older people in an appropriate manner, as older people may lack confidence when first using this technology. To a large extent, tablet learning has developed outside of formal education; however, informal learning may not be appropriate for older people who typically have more limited exposure to computer technology. Formal tablet training may introduce technology into older people's lives in an accessible way, and assist them in keeping up-to-date with technological advances and current trends. Czaja et al. (2012) evaluated a community-based computer and internet training program designed for older adults and concluded that it can be effective in terms of increasing computer and internet skill as well as help older adults become more comfortable with computer technology. Ultimately, older people might enjoy the advantages that new technologies can offer, such as quick access of information and social inclusion (Warschauer, 2004; Morris, 2013).

Overall, the current results are consistent with the Selection Optimization Compensation (SOC) Theory which postulates that there are three fundamental life management processes: selection (goals), optimization (goal-related means to achieve desired goals) and compensation (reaction to loss in goal-related means to maintain success or desired goals) (Baltes, 1997). According to SOC, as people grow older, they allocate more resources toward loss management to be able to maintain their goals, which reflects compensation to maintain stability (Baltes, 1997). Although the current study did not directly investigate whether participants selected goals, and whether they compensated to maintain these goals, our participants emphasized the importance of learning new things, and keeping up-to-date with current trends (e.g., “I just think we're like a forgotten generation, that's what I feel like. You want to go in and you want to be able to talk with your family and your grandchildren and not look vacant when they say, I'm going to do this.”).

Participants also showed evidence of optimizing behavior, such as requesting or accepting assistance to use technology to achieve their desired goals (e.g., “I just go to my son or my daughter if I need something that I can't get anywhere else and they'll do it for me”). This example of behavior further supports SOC which posits that older adults require more time, practice and cognitive support to achieve learning gains. In addition, our results are consistent with the Adult Learning Theory (Knowles, 1984). One of the principles of this theory is that adults need to be involved in the planning and evaluation of their instruction. Our participants expressed frustration when they requested assistance and other people completed the job for them rather than providing guidance. This is relevant to another principle of the Adult Learning Theory in which experience provides the basis for the learning activities. Completing tasks for older adults may not only be a source of frustration for them but represents the loss of an opportunity for them to gain experience and learn a new activity.

Our findings may be used to inform technology developers and manufacturers about tablet refinement, thereby increasing the potential for acceptance and adoption of tablets by older adults. Several tablet features are worth consideration. For example, the buttons and keyboard should be larger with clear indication of their function. The larger tablets that we included weighed over 500 g and were felt to be heavy; therefore, tablet weight appears to be a consideration for older people, and while our findings might suggest not exceeding 500 g, specific product testing with this age group would be justified. Our participants did not reach consensus in terms of size. Some participants noted that the smaller tablets were too small to see the screen, and others that the larger tablets were too large to have on them at all times. Another point for refinement is related to operational guidance. Participants felt that instructions are typically difficult for non-technical people to understand. Unlike other electronic equipment (e.g., digital cameras), instructions are typically not included with tablets. Manufacturers might include hardcopy instructions that clearly explain basic functions of a tablet, such as the location of the on/off button and how this is used. For example, in some cases the power button must be held for a few seconds to turn a tablet off whereas others require a single quick press.

We should point out that the majority of participants were female, and therefore, our findings may not transfer to males. Despite that, the gender imbalance may reflect current societal trends. Previous studies found that males are more likely to use or own technological equipment compared with females (Wilson et al., 2003; Pinkard, 2005). Therefore, it is likely that fewer males were tablet novices, and therefore, eligible to participate in our study. Survey studies could provide insight into whether the gender imbalance in our study was due to fewer females using tablets than males, or due to other reasons such as females being keener to volunteer for research purposes. Although our sample was small, and predominantly female, the quotes presented suggest that the responses were similar for men and women; while consistent, a larger sample of men would be necessary to more fully compare the similarities and differences by gender. Previous studies have, for example, reported gender differences. Czaja et al. (2006) found that older women used fewer types of technology, were more anxious and had less positive general attitudes about computers relative to older men. Czaja and Sharit (1998) reported that women found computers more dehumanizing following task experience; however, women also experienced a greater sense of comfort following task experience compared with men.

The inductive nature of analysis was maintained in the sense that there was genuine interest in the raw data to reveal any themes; however, our engagement with previous research, which we aimed to build upon, led to a familiarization with certain concepts, a “conceptual organization.” This can be congruent with thematic analysis, as suggested by Boyatzis (1998). Sets of underlying ideas and themes in the relevant literature, such as “challenges” and “barriers” relating to the use of tablets, were carefully recorded, and were brought into the analysis. They were, specifically, used during the process of clustering the subthemes in the later stages of the analysis, serving as a guide for developing “meaningful” higher simple themes. It is therefore the subthemes which appear under the “higher simple” themes which explicate the findings of this study.

Our sample included only young-old (i.e., 65–75 years) individuals. The age range was restricted to help control potential cohort effects, and to be in line with inclusion criteria of the following stage of the study. Moreover, the majority of participants were White British, so our sample lacked ethnic diversity. Despite that our results are comparable to Heinz et al. (2013) who used a midwestern USA sample, suggesting that our results could be transferred beyond the UK. Nevertheless, certain socio-economic variables, such as education, may be higher in our sample compared to other populations (Anderson et al., 2004). Finally, our sample was small and therefore larger studies are needed to be able to generalize these findings to populations.

The focus of the current study was on naïve participants, as in the proposed tablet intervention, participants will be using tablet technology for the first time. Despite that, the views of more experienced tablet users would be interesting, and further work is planned post-intervention (Vaportzis, under review). Finally, although the participants enjoyed the tablet experience and emphasized the likelihood of using a tablet in the future, these results should be interpreted with caution given the possible response bias of participants in the focus group setting. While selection bias was not actively prevented, one third of the participants reported that they were undecided or unlikely to use a tablet in the future, suggesting that it was not only people who were interested in using tablets participated.

In conclusion, our findings suggest that the majority of our participants were eager to adopt new technology and willing to learn using a tablet. However, concern about the process of learning was noted. Participants voiced apprehension about lack or unclear instructions and support. Understanding older adults' perceptions of technology is vital to assist with introducing technology to this population and maximize the potential of technology to facilitate independent living. Future studies investigating older adults' attitudes toward technology and tablets, and their potential to improve health and wellbeing in older adults are warranted and represent a strong research framework.

## Author contributions

EV conceptualized and designed the study; acquired, analyzed, and interpreted the data; and drafted the manuscript. MGC analyzed and interpreted the data. AG conceptualized and designed the study. All authors revised the manuscript critically for important intellectual content; gave final approval of the version to be published; and agreed to be accountable for all aspects of the work in ensuring that questions related to the accuracy or integrity of any part of the work are appropriately investigated and resolved.

### Conflict of interest statement

The authors declare that the research was conducted in the absence of any commercial or financial relationships that could be construed as a potential conflict of interest. The reviewer, SL, declared a shared affiliation, with no collaboration, with one of the authors, AG, to the handling Editor.
